# Three new records of Nannosquillidae from Taiwan with notes on their ecology (Crustacea, Stomatopoda, Lysiosquilloidea)

**DOI:** 10.3897/zookeys.721.20588

**Published:** 2017-12-12

**Authors:** Jing-Wen Wang, Tsyr-Huei Chiou

**Affiliations:** 1 Department of Life Sciences, National Cheng Kung University, Tainan 70101, Taiwan

**Keywords:** Kenting National Park, Nannosquillidae, new record, *Pullosquilla*, western Pacific

## Abstract

The genus *Pullosquilla* Manning, 1978, including *P. litoralis*, *P. thomassini*, and *P. pardus*, has been found in Taiwan for the first time. All three species live in a subtidal sand flat north of the Bitou fishing port within the Kenting National Park, Taiwan. Adult specimens were examined, illustrated, and photographed. The habitat, which all three species share, is described. The implication of such closely related species sharing the same habitat is discussed.

## Introduction

Among the fourteen genera in the family of Nannosquillidae Manning, 1980, nine have been found in the Indo-West Pacific region (Ahyong 2001). However, only two genera, *Acanthosquilla* and *Bigelowina*, are currently known from Taiwan (Ahyong et al. 2008). Located near the center of Indo-West Pacific region, the number of taxa of stomatopod crustaceans found around Taiwan is surprisingly low (Yeh and Hsueh 2010). One possible explanation is that species with small adult size (e.g., smaller than 5 cm) or with deep burrows could have slipped through traditional collecting gears such as trawl nets or shrimp pots. Here, a detailed visual search was conducted on a subtidal sand flat and recorded, for the first time, three species of *Pullosquilla* Manning, 1978 from Taiwan.

## Materials and methods

Surveys of sand-dwelling stomatopods were conducted in April 2014, November 2014, and June–July 2017. All specimens were collected in a sand flat north of the Bitou fishing port in the Kenting National Park, Pingtung County, Taiwan. The approximate GPS coordinates are 21°54.69'N, 120°50.76'E. The sampling site, between 5 to 7 m under water, has an undulating white sandy substrate. Specimens were found by visually locating their burrow entrances, usually a pair of circular holes 2–4 mm in diameter. Using a hand net to trawl 1–2 L of sand, the animal was sieved out on the spot. While sieving, special attention was given to avoid a sandy plume rising above the rim of the hand net; shaking the hand net horizontally for a few seconds at a time and looking for the animals above the remaining sand. Once found, individuals or pairs of animals were placed in 20 ml plastic vials with ambient seawater and brought back to laboratory for identification. Collected specimens were measured, photographed, and transferred to 75% alcohol for further analysis. Examined specimens in this study are deposited in National Museum of Nature Science (**NMNS**), Taichung City, Taiwan and National Chung Kung University (**NCKU**), Tainan City, Taiwan.

Morphological terminology and abbreviations follow Ahyong (2001). Total length (TL) was measured along the midline from apex of the rostral plate to the apices of the submedian teeth. Abbreviations include:

**A1** antennule

**A2** antenna

**AS** abdominal somite

## Taxonomy

### Pullosquilla litoralis

Taxon classificationAnimaliaORDOFAMILIA

(Michel & Manning, 1971)

Figs 1 , 2 , 3

Austrosquilla litoralis Michel & Manning, 1971: 237–239, fig. 1.
Pullosquilla litoralis : Manning 1978: 19–20; Ahyong 2001: 165–166, fig. 82.

#### Material examined.

NMNS-7834-001, 1 female (TL 11.8 mm), April 2014. NCKU-0103-01, 1 male (TL 13.1 mm); NCKU-0103-02, 1 female (TL 8.8 mm), November 2014. NMNS-7834-002, 1 female (TL 15.7 mm); NMNS-7834-003, 1 male (TL 13.7 mm); NMNS-7834-004, 1 male (TL 18.0 mm), July 2017.

#### Diagnosis.

Cornea subglobular. Eyes extending to the end of the A1 peduncle. Rostral plate with acute apex; triangular-shaped, broader than long. Ocular scale fused along midline. Dorsal processes of A1 somite forming long triangular lobes directed anterolaterally (Fig. 2A).

**Figure 1.**
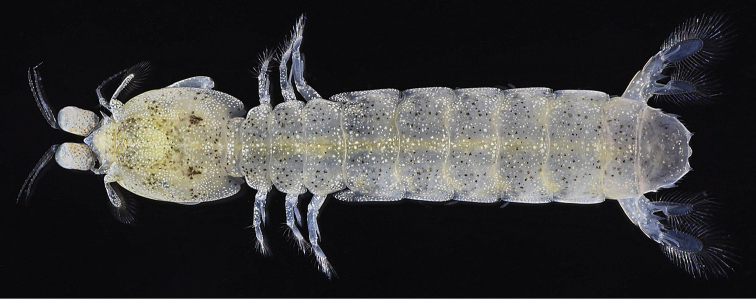
*Pullosquilla litoralis* Michel & Manning, 1971, male specimen from Taiwan. TL 18.0 mm, dorsal view, color in life.

**Figure 2.**
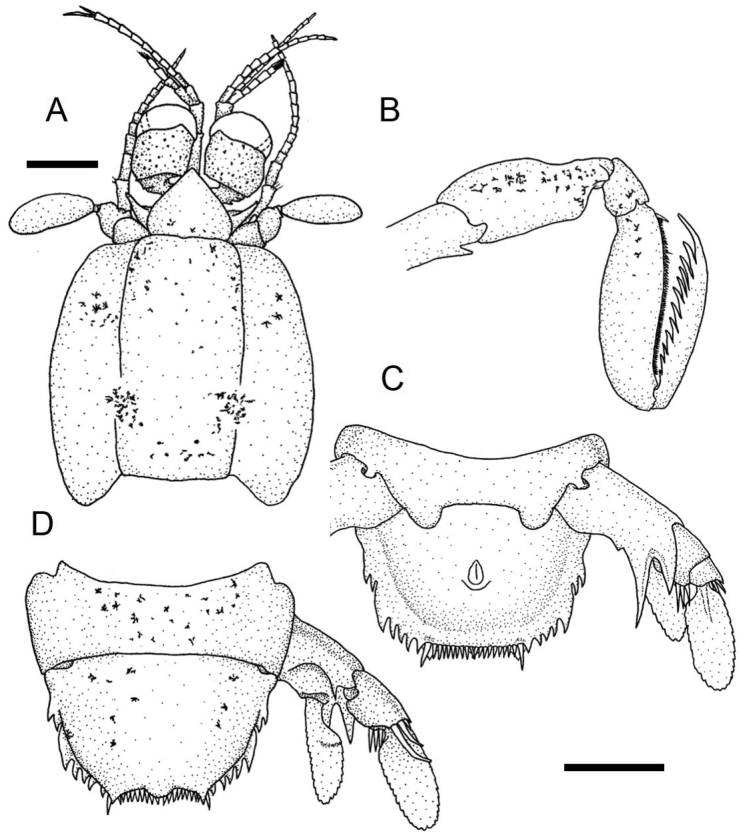
*Pullosquilla litoralis* Michel & Manning, 1971, male specimen from Taiwan, TL 18.0 mm: **A** cephalon, dorsal **B** raptorial claw, right lateral **C** telson and uropod, ventral **D** telson and uropod, dorsal. Scale bars: 1 mm.

Rostral claw dactylus with 10–12 teeth. Propodus occlusal margin pectinate, with four movable spines proximally. Distal end of ischium ventrally armed with a short spine (Fig. 2B). Basal segments of pereiopods 1–3 with short lateroventral spine. Mandibular palp absent; five epipods present.

AS6 without ventrolateral spine anterior to the uropod articulation (Fig. 2C). Telson with median semi-circular projection and submedian projection, acute in males greater than 13 mm but blunt in females. Posterior margin of telson with one pair of movable submedian teeth and 7–9 submedian denticles on either side of midline. Posterolateral margins of telson with two pairs of fixed primary teeth, of which lateral primary teeth are smaller than intermediate ones, and with one lateral and four intermediate denticles (Fig. 2D).

Uropodal protopod with single distal spine at inner margin above articulation of exopod; inner primary spine longer than outer. Outer margin of proximal uropodal exopod segment with three curved, movable spines, inner margin with 2–4 stiff setae. Exopod distal segment ovate and elongated. Endopod subtriangular and elongated (Fig. 2C, D).

#### Distribution.

French Polynesia and Australia to the Western Indian Ocean (Ahyong 2001), and now Taiwan. Currently, Taiwan is the northernmost habitat known for *P. litoralis*.

#### Remarks.

**Figure 3.**
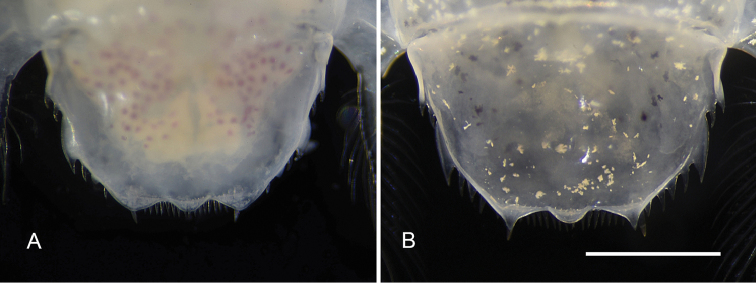
Morphology of telson in female and male *Pullosquilla litoralis* Michel & Manning, 1971: **A** female telson, dorsal view, TL 15.7 mm **B** male telson, dorsal view, TL 18.0 mm. Scale bar: 1 mm.

The specimens of *P. litoralis* from Taiwan agree well with the female holotype reported in Michel and Manning (1971) and other collections described in Manning (1978) and Ahyong (2001). In addition to sexual dimorphic coloration in their chromophores (Ahyong 2001), it was observed that the shape of submedian projections on the telson also differ between sexes. By comparing the adult male and female specimens with TL greater than 13 mm, it is noticeable that the posterior margin form blunt and short submedian projections in females, while males bear acute and long submedian projections, which protrude beyond the median lobe of the telson (Fig. 3).

### Pullosquilla thomassini

Taxon classificationAnimaliaORDOFAMILIA

(Manning, 1978)

Figs 4 , 5

Pullosquilla thomassini Manning, 1978: 20–21, fig. 9; Manning 1980: 269–270; Ahyong 2001: 168–170, fig. 84.

#### Material examined.

NMNS-7834-009, 1 female (TL 14.6 mm), June 2017. NMNS-7834-010, 1 male (TL 16.2 mm), July 2017.

#### Diagnosis.

Cornea subglobular. Eyes reaching to the end of the A1 peduncle. Rostral plate triangular, longer than broad; apex depressed anteriorly. Dorsal processes of A1 somite forming sharp spines directed anterolaterally; with slightly sinuate margin. A2 protopod with blunt projection adjacent to basal rostral plate (Fig. 5A).

**Figure 4.**
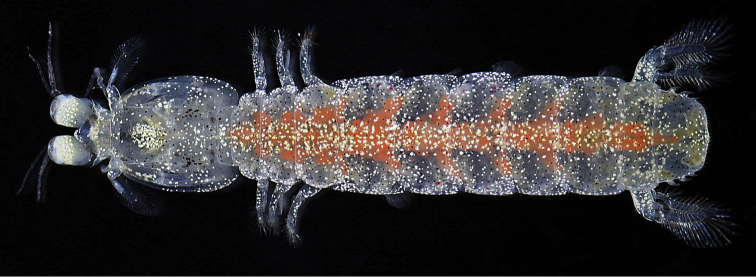
*Pullosquilla thomassini* Manning, 1978, female specimen from Taiwan. TL 14.6 mm, dorsal view, color in life.

**Figure 5.**
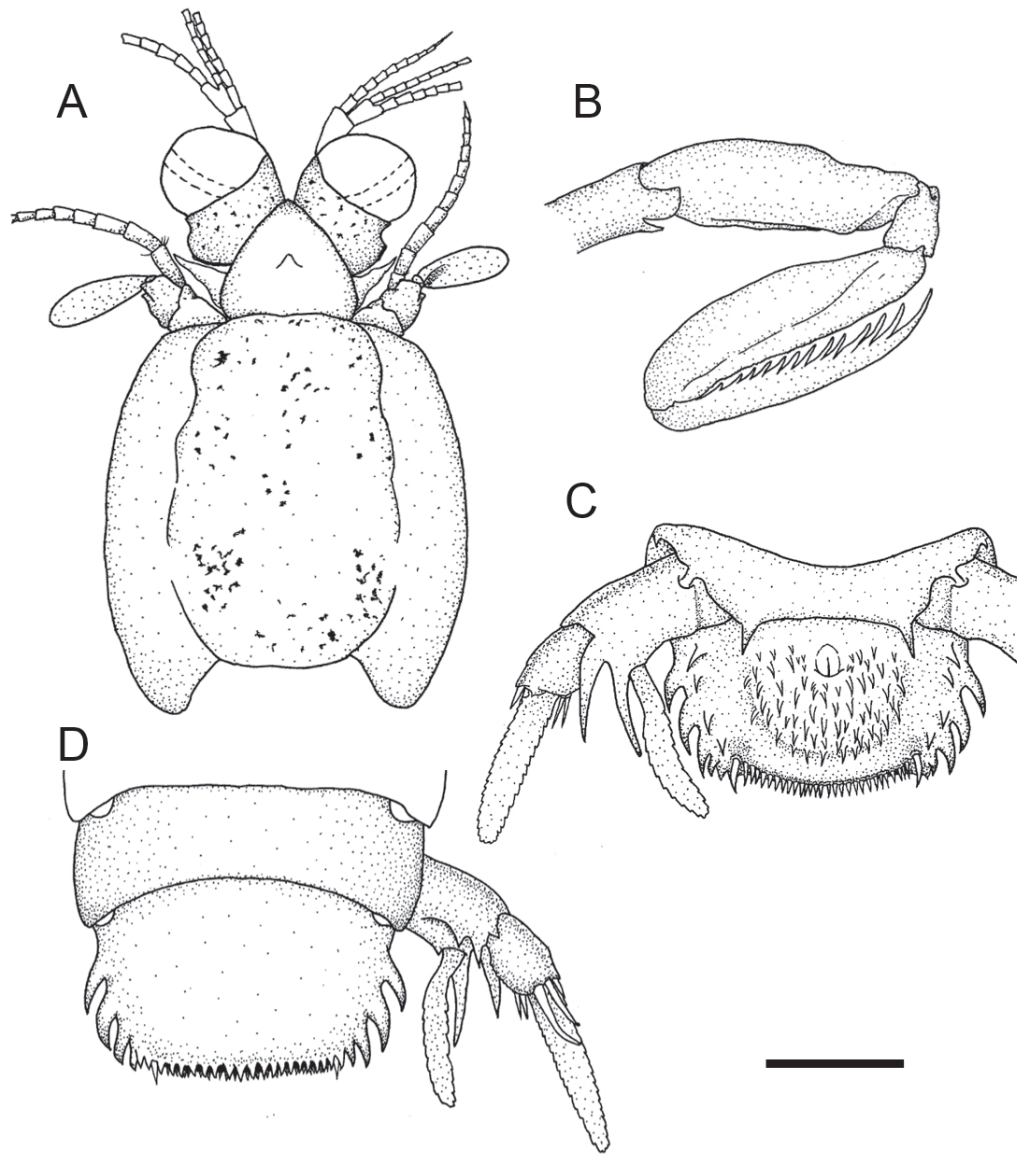
*Pullosquilla thomassini* Manning, 1978, female specimen from Taiwan, TL 14.6 mm: **A** cephalon, dorsal **B** raptorial claw, right lateral **C** telson and uropod, ventral **D** telson and uropod, dorsal. Scale bar: 1 mm.

Rostral claw with 13–14 teeth on dactylus. Propodus pectinate; occlusal margin pectinate, and with 4 movable spines proximally. Distal end of ischium ventrally armed with slender spine (Fig. 5B). Basal segment of pereiopods 1–3 posteriorly with short spine. Mandibular palp absent; five epipods present.

AS6 with one ventrolateral spine at each lateral margin anterior to uropodal articulation; posterior margin on ventral surface with two spines directed posteriorly. Telson broader than long; dorsal surface smooth; mid-ventral surface covered with short spines. Lateral margin of telson with four broad and curved spines directed posteriorly, each ventro-medially flanked by slender spine. Posterior margin of telson depressed, forming a broad false eave with 21 posteriorly directed small spines, placed in row. Ventromedial telson margin with 12–13 submedian denticles either side of midline (Fig. 5C, D).

Uropodal protopod with two distal spines above proximal exopod articulation; with two primary spines, inner spine longer than outer spine. Outer margin of uropodal exopod proximal segment with 3 movable spines, inner margin with 2–4 stiff setae. Distal segment of exopod and endopod slender and elongated (Fig. 5C, D).

#### Distribution.

Based on Ahyong (2001), *P. thomassini* is widely distributed in the Indo-West Pacific region (Australia, French Polynesia to Ogasawara Island, Japan). These specimens are the first records of *P. thomassini* from Taiwan.

#### Remarks.

The telson of *P. thomassini* is unique in the genus by bearing four strong spines on lateral margins, each flanked by a short spine on its inner ventral surface (Fig. 5C). Two specimens examined here, a female (TL 14.6 mm) and a male (TL 16.2 mm), agree well with the descriptions of *P. thomassini* by Manning (1978) and Ahyong (2001).

### Pullosquilla pardus

Taxon classificationAnimaliaORDOFAMILIA

(Moosa, 1991)

Figs 6 , 7

Pullosquilla pardus Moosa, 1991: 184–185, fig. 8; Ahyong 2001: 165–168, fig. 83.

#### Material examined.

NCKU-0102-01, 1 male (TL 12.3 mm); NCKU-0103-03, 1 male (TL 17.9 mm); NCKU-0103-04, 1 female (TL 13.1 mm), November 2014. NCKU-0104-01, 1 female (TL 19.7 mm); NCKU-0104-02, 1 female (TL 22.7 mm); NCKU-0104-03, 1 female (TL 21.9 mm); NCKU-0104-04, 1 male (TL N/A); NCKU-0104-05, 1 male (TL 23.1 mm); NMNS-7834-005, 1 male (TL 21.3 mm); NMNS-7834-006, 1 male (TL 20.4 mm); NMNS-7834-007, 1 female (TL 23.8 mm), June 2017. NCKU-0105-04, 1 female (TL N/A); NCKU-0105-05, 1 female (TL 24.0 mm); NCKU-0105-06, 1 female (TL 20.9 mm); NMNS-7834-008, 1 female (TL 25.3 mm); NCKU-0105-08, 1 female (TL 19.4 mm); NCKU-0105-09, 1 female (TL 23.2 mm); NCKU-0105-10, 1 female (TL 22.8 mm), July 2017.

#### Diagnosis.

Cornea subglobular. Eyes reaching to end of A1 peduncle. Rostral plate cordiform, broader than long. Dorsal processes of A1 somite forming elongated triangular lobes directed anteriorly. A2 protopod with mesial and ventral papilla (Fig. 7A).

**Figure 6.**
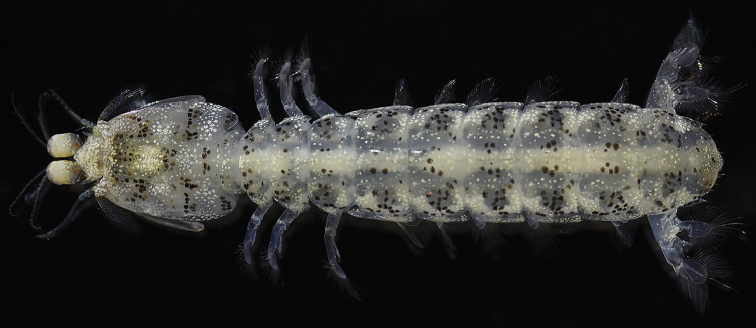
*Pullosquilla pardus* Moosa, 1991, male specimen from Taiwan. TL 20.4 mm, dorsal view, color in life.

**Figure 7.**
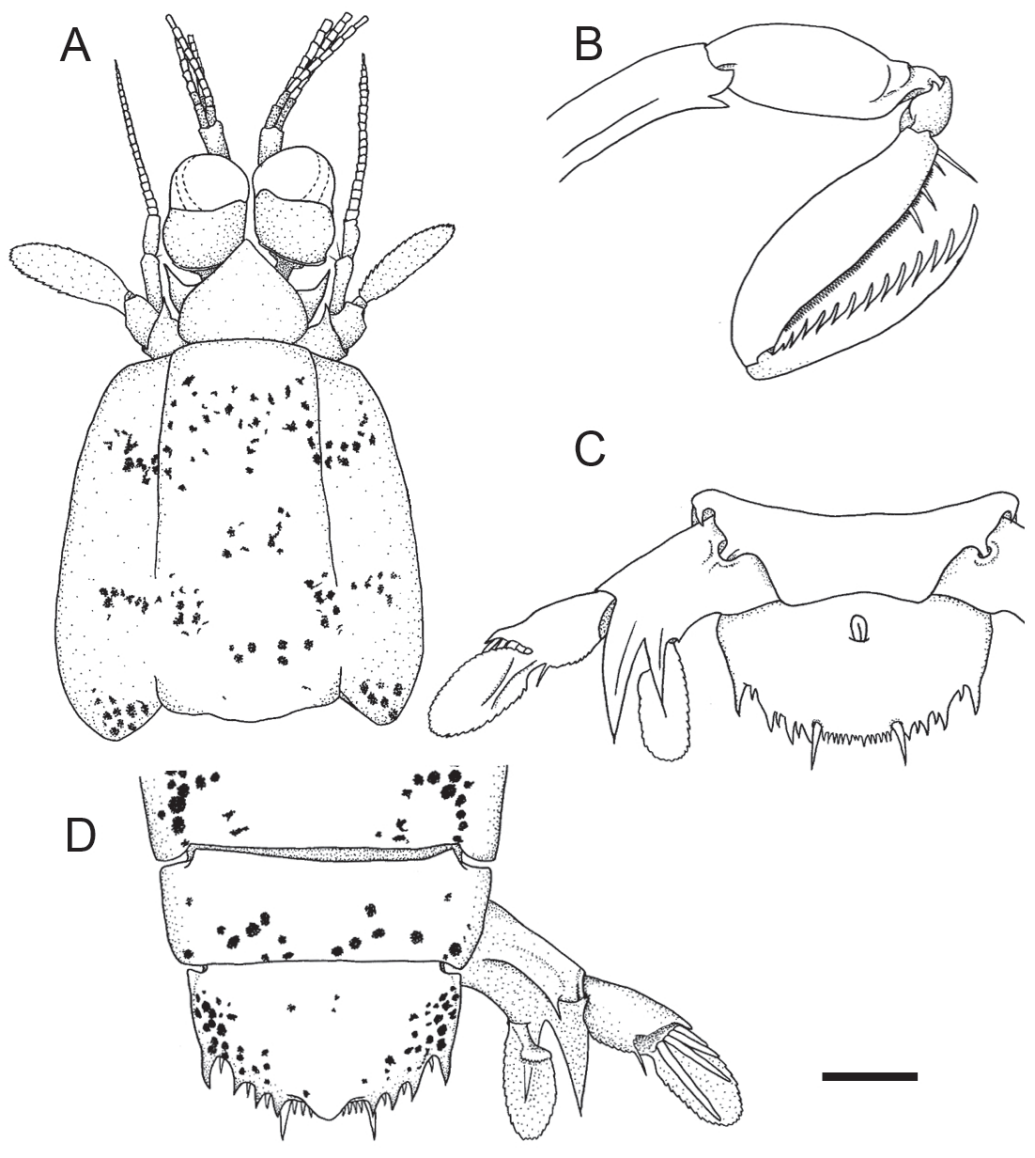
*Pullosquilla pardus* Moosa, 1991, male specimen from Taiwan, TL 21.3 mm: **A** cephalon, dorsal **B** raptorial claw, right lateral **C** telson and uropod, ventral **D** telson and uropod, dorsal. Scale bar 1 mm.

Raptorial claw dactylus with 12–13 teeth; propodus occlusal margin pectinate, proximally with 4 movable spines. Distal end of ischium armed with an anteroventrally-directed spine (Fig. 7B). Basal segment of pereiopods 1 and 2 each armed with inner and outer spines; basal segment of pereiopod 3 with outer spine only. Mandibular palp absent, five epipods present.

AS6 with ventrolateral spine anterior to uropodal articulation (Fig. 7C). Telson broader than long; dorsal surface posteriorly with one blunt subtriangular projection; lateral margins unarmed; posterior margin with a pair of movable submedian teeth and four pairs of fixed primary teeth. Margins between each primary tooth with denticle except for submedian margin, with 5–8 submedian denticles either side of midline (Fig. 7D).

Uropodal protopod with slender distal spine at inner margin above articulation of exopod. Outer margin of proximal uropodal exopod segment laterally armed with a short fixed spine; distally with4 straight movable spines directed posteriorly; medially with stiff seta. Exopod distal segment and endopod both ovate in shape (Fig. 7C, D).

#### Distribution.

New Caledonia through Timor Sea to northwest shelf, Australia, and now Taiwan. This is the first record of *P. pardus* in the northern Hemisphere.

#### Remarks.

According to Moosa (1991), *P. pardus* most closely resembles *P. malayensis* of the other *Pullosquilla* species (Moosa 1991). Specimens collected in this study agree well with the characteristics of *Pullosquilla pardus* reported in Moosa (1991) and Ahyong (2001). In addition to the differences described by Moosa (1991), the outer spine of the uropodal protopod in our specimens is always longer and stronger than the inner spine, unlike that in *P. malayensis*, which has a shorter and slenderer outer spine (Manning 1968).

## Discussion

*Pullosquilla* is a genus of small sand burrowing stomatopods within the superfamily Lysiosquilloidea. Currently, *Pullosquilla* contains four species: *P. litoralis*, *P. thomassini*, *P. pardus*, and *P. malayensis* (Ahyong 2001). They grow into one of the smallest adults among spearing stomatopods and are named for their immature appearance (Manning 1978). Similar to many other Lysiosquilloidea, they often live in U-shaped burrows made of a mucus-sand mixture (pers. obs.; Caldwell 1991). However, within the Nannosquillidae, *Pullosquilla* is the only genus known to form long-term mated pairs sharing the same burrow (Jutte 1997; Wright 2013). As a result of this special monogamous life style, *P. litoralis* and *P. thomassini* has been systematically studied with respect to their pairing and biparental care behavior (Jutte 1997; Lindstrom 2003; Wright and Caldwell 2015).

Probably due to the scarcity of specimens, the pairing and breeding systems of *P. pardus* have rarely been studied. The only report regarding monogamous pairing of *P. pardus* is speculation on taxonomic samples collected from Timor Sea (Ahyong 2001). In the current study site, more than half of the burrows located belonged to *P. pardus.* Likewise, monogamous pairs were extracted from single U-shaped burrows, some of which were still caring for their donut-shaped egg clutch. Consequently, monogamous pair bonding and parental care in *P. pardus* is now confirmed.

In this study, only a tiny portion, approximately 150 m^2^, of a relative large and continuous sandy bottom of more than ten hectares were surveyed. In such a small area, three of the four known *Pullosquilla* species, *P. thomassini*, *P. litoralis*, and *P. pardus*, were discovered. Although we failed to find *P. malayensis*, it is interesting to note that this is the first record of the sympatry of more than two *Pullosquilla* species. The previous records regarding the co-occurrence of *Pullosquilla* came from Tiahura, Moorea, Society Islands, and Tulear, Madagascar, where only *P. litoralis* and *P. thomassini* could be found from the surveyed reef complexes (Manning 1978). However, based on the time, depth, and location of collection it is highly possible that *P. malayensis* and *P. thomassini* coexist in Double Reefs, Guam (Ahyong and Erdmann 2003).

The study site is located slightly south of the famous Shell Beach Preservation Zone of the Kenting National Park. While it might appear to be a uniform continuous sandy substrate, there could be some unobserved factors that support the coexistence of these *Pullosquilla* species. One particular feature of the collection site, which is also the reason for its designation of the Shell Beach Preservation Zone, is the composition of the sand. From the slope of the Shell Beach, it is immediately obvious that the sand grains are very coarse. In conjunction with the nearshore currents, the sandy seabed rarely settles. As a result, there is hardly any coral or algae cover beyond the drop-offs of the nearshore reef flat. The lack of hiding places or food for relatively larger predators in this region could thus be favored by these small sand-dwelling stomatopods.

No research on *P. pardus* has been done beyond its taxonomic descriptions, probably due to few accessible localities were known for surveys. In Moosa (1991) and Ahyong (2001), among four records of the *P. pardus*, the holotype of *P. pardus* and one of the specimens from Australia were both collected at depth over 40 m, while only two other specimens were captured in sand at 6 m. Here, in this survey, more *P. pardus* were caught than *P. litoralis* and *P. thomassini* combined: of the 24 burrows, 17 belonged to *P. pardus* while only five and two burrows were occupied by *P. litoralis* and *P. thomassini*, respectively. Among them, two monogamous pairs were evacuated from two burrows, one of which was occupied by *P. pardus* and the other belonged to *P. litoralis*. No triples were found in any of the burrow. During the collection, *P. pardus* could easily be recognized by its panther-like color pattern and large body size, while other two species needed further examination. Although the sample sizes are too small to have any statistical significance, during the survey it was observed that *P. pardus* were evenly distributed throughout the subtidal region. They could be caught from near shore sandy substrate at approximately 3 m depth to the sandy plain at 7–9 m depth. On the other hand, the burrows occupied by *P. litoralis* were usually found on the sandy plain farther away from shore, at depths of 6 to 9 m. They were rarely observed on the near shore sandy substrate at 3-5 m depth. Although our sampling did not cover all seasons, it is suggested that the current location could be a good locality for a detailed study of the behavior of *P. pardus*. Except for the genus *Pullosquilla*, several burrows of larger stomatopods were also observed, potentially those of Lysiosquilloidea, on the same sand plain. However, proper collecting tools are required in order to retrieve these larger stomatopods for further examination and identification.

Stomatopod crustaceans are abundant worldwide throughout tropical and temperate waters, where they burrow in coral rock, coral rubble, or sandy benthic substrates (Ahyong et al. 2017). There were 29 genera, and 64 species of stomatopod crustaceans reported from Taiwan (Ahyong et al. 2008; Yeh and Hsueh 2010). In this study, by performing detailed hand surveys throughout the subtidal sand flat, we have successfully found new records from Taiwan, including an additional genus *Pullosquilla* and three more species of *Pullosquilla*. Since previous studies obtained specimens mainly by trawling, certain species were inevitably under-represented. For example, the records of Lysiosquilloidea were limited, presumably because larger species typically burrow too deeply while smaller species are too small to be caught in trawl nets. Overall, our findings not only expand the reported distribution of *Pullosquilla* stomatopods in the western Pacific Ocean but also contribute to the understanding of cohabitation and monogamous behaviors in genus *Pullosquilla*. Further studies comparing mating and breeding behaviors among these species will likely yield more insight into their unique monogamous system. Based on the diversity of stomatopods found in the Indo-West Pacific region, it is assumed that numerous stomatopod species are yet to be discovered from Taiwan.

## Supplementary Material

XML Treatment for Pullosquilla litoralis

XML Treatment for Pullosquilla thomassini

XML Treatment for Pullosquilla pardus
